# Supplementary material for: CD206+ Trem2+ macrophage accumulation in the murine knee joint after injury is associated with protection against post-traumatic osteoarthritis in MRL/MpJ mice
Source: PLoS One. 2025 Jan 3;20(1):e0312587. doi: 10.1371/journal.pone.0312587 (PMC11698337; doi:10.1371/journal.pone.0312587)
Supplement: S1 File — (PDF) [file pone.0312587.s001.pdf]

CD206+ Trem2+ Macrophage Accumulation in the Murine Knee Joint After Injury is Associated with Protection Against Post-Traumatic Osteoarthritis in MRL/MpJ Mice

Jillian L. McCool[1,2], Aimy Sebastian[1], Nicholas R. Hum[1], Stephen P. Wilson[1], Oscar A. Davalos[1], Deepa K. Murugesh[1], Beheshta Amiri[1], Cesar Morfin[1,3], Blaine A. Christiansen[3], Gabriela G. Loots[1,2,3*]

[1]*Lawrence Livermore National Laboratory, Physical and Life Science Directorate, Livermore, CA.*

[2]*University of California Merced, School of Natural Sciences, Merced, CA.*

[3]*University of California Davis Health, Department of Orthopaedic Surgery, Sacramento, CA.*

Supplementary Figures and Figure Legends

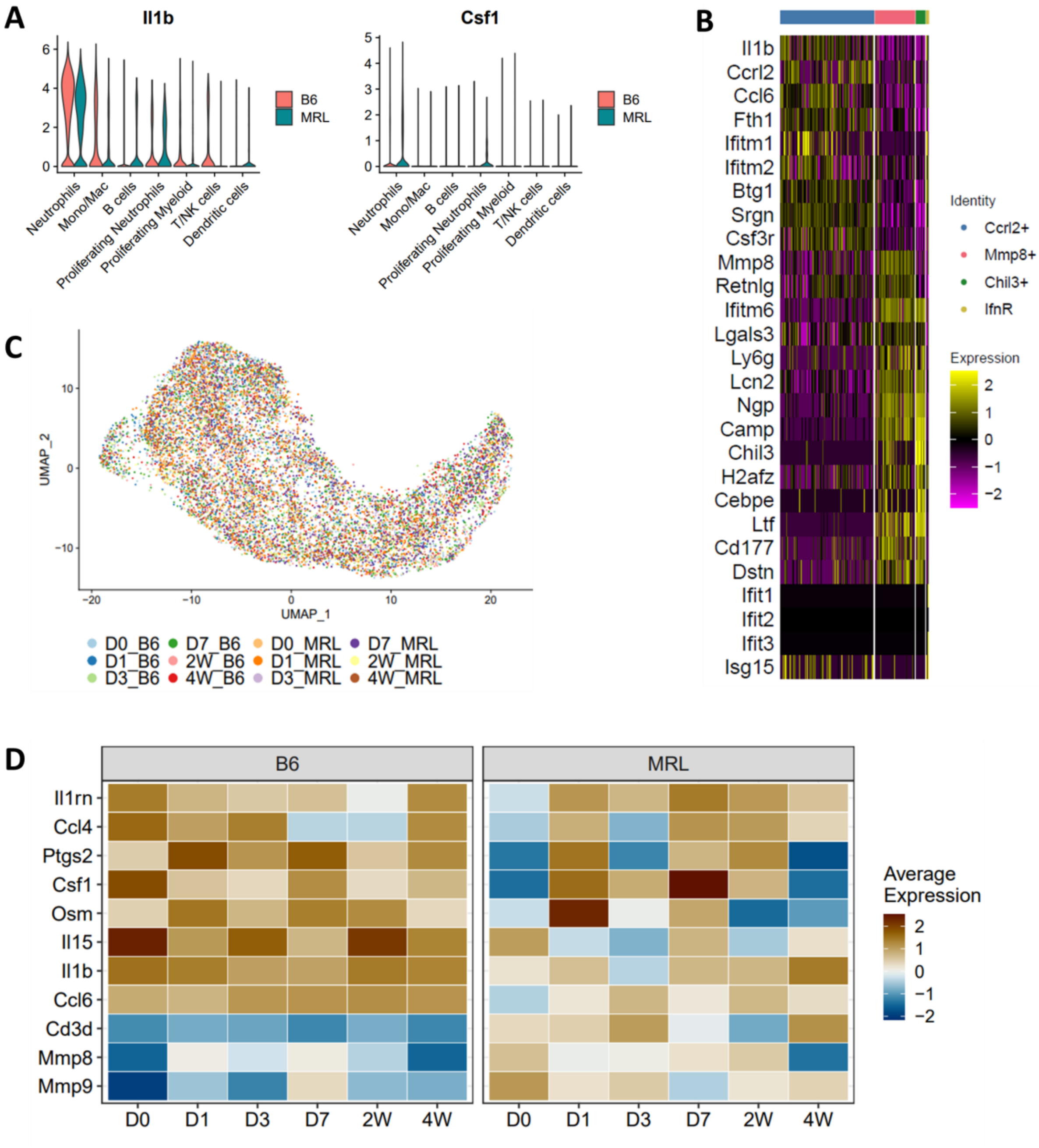

Figure S1. Characterization of transcriptional changes in neutrophils after knee joint injury, in MRL and B6 mice. *A) Neutrophils show enrichment for Il1b, a key cytokine implicated in osteoarthritis pathogenies and Csf1, a key regulator of monocyte to macrophage differentiation. B) Heatmap showing the expression of key neutrophil subtype markers. C) UMAP plot showing the overlay of neutrophils from various experimental groups. D) Heatmap showing a subset of genes differentially expressed between B6 and MRL neutrophils.*

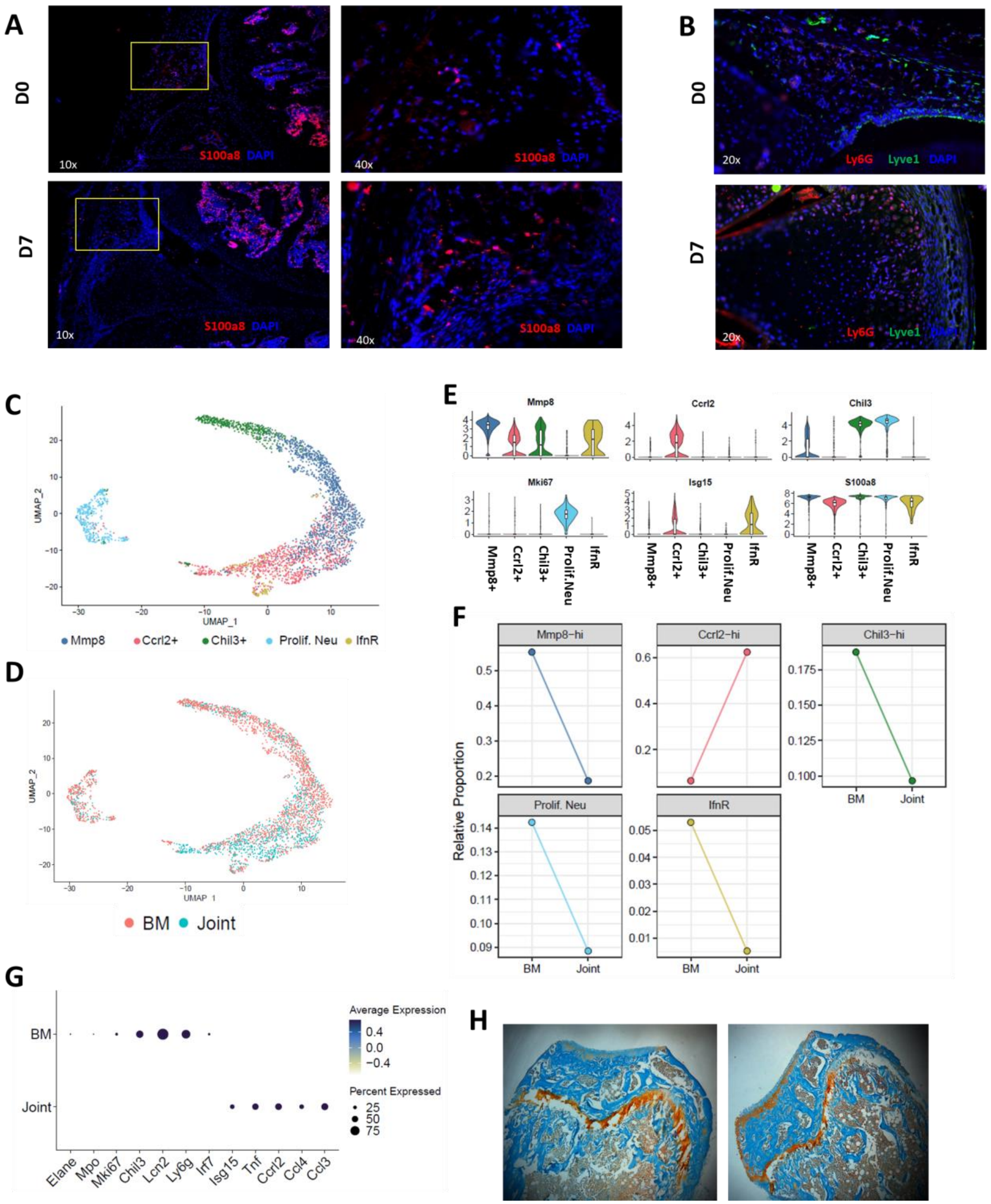

Figure S2. Comparison of neutrophils from the knee joint and bone marrow. *A) IHC showing infiltration of S100a8+ cells in B6 knee joint after injury. Regions in the yellow boxes are shown at a higher magnification on the right side of the panel. B) IHC showing the expression of Lyve1, a marker of synovial lining macrophages and Ly6g, a marker of neutrophils, in B6 knee joint at*

D0 and D7. More Ly6g+ cells were observed in D7 joints. C) UMAP plot showing the overlay of bone marrow and joint neutrophils, colored by neutrophil subclusters. D) UMAP plot showing the overlay of bone marrow and joint neutrophils, colored by experimental groups. E) Violin plots showing key neutrophil subtype markers in integrated bone marrow and joint neutrophil scRNA-seq data F) Graph showing the differences in the proportion of various neutrophil subtypes in the bone marrow and the joint. G) Dot plot showing a subset of genes differentially expressed between bone marrow- and joint neutrophils. H) Safranin-o and fast green staining of the digested limbs. The digestion protocol was optimized to release cells from soft tissues in the joint without bone marrow contamination. The bone remained intact after the digestion.

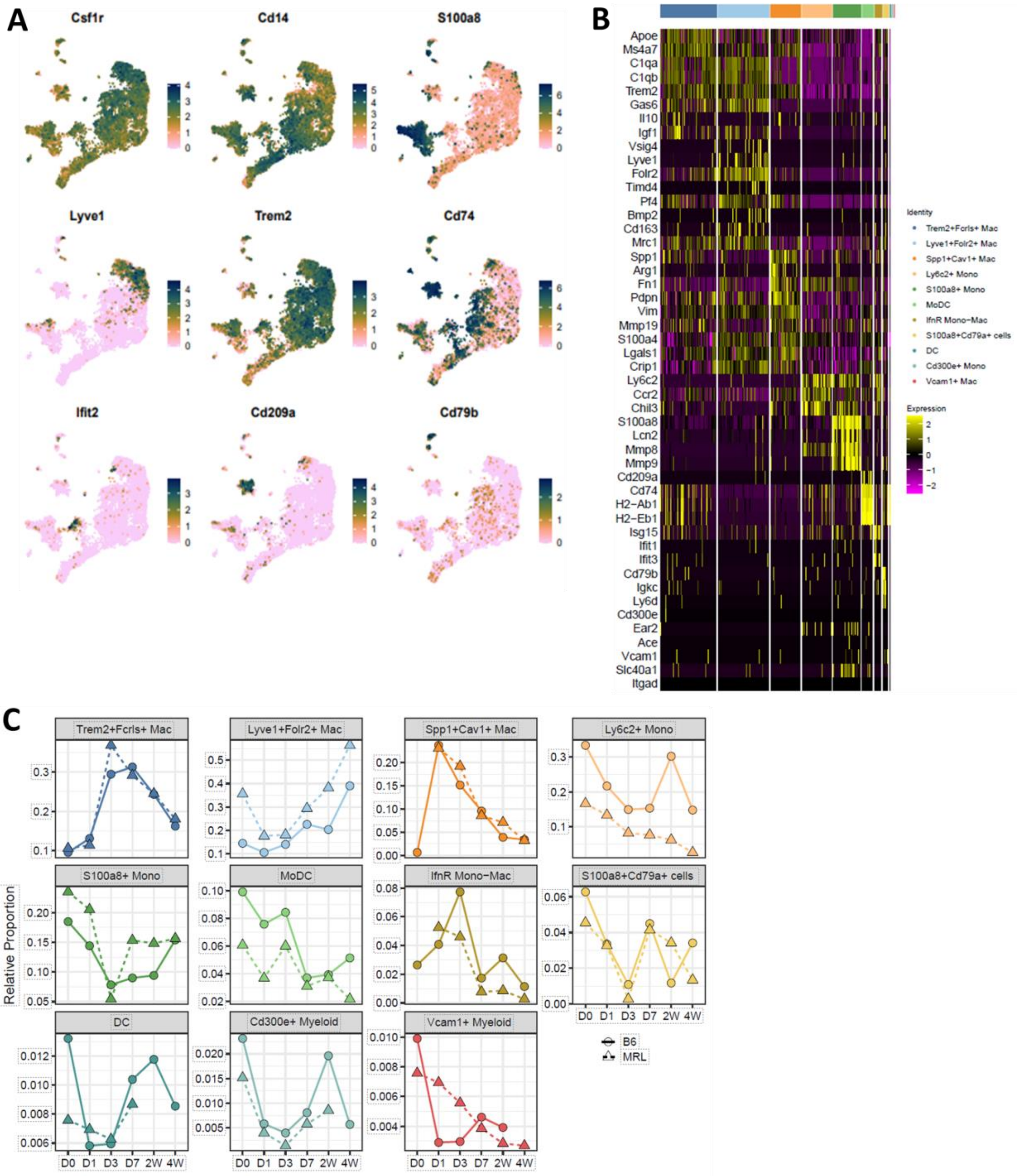

Figure S3. Single-cell level profiling of Mono/Mac subpopulations from MRL and B6 knee joints. *A) Feature plots showing the expression of key markers of monocyte and macrophage subpopulations. B) Heatmap showing a subset of genes enriched in each monocyte and macrophage subcluster relative to other Mono/Macs. C) Proportion of each monocyte and macrophage subpopulation relative to total Mono/Macs sequenced at each timepoint, in MRL and B6 mice.*

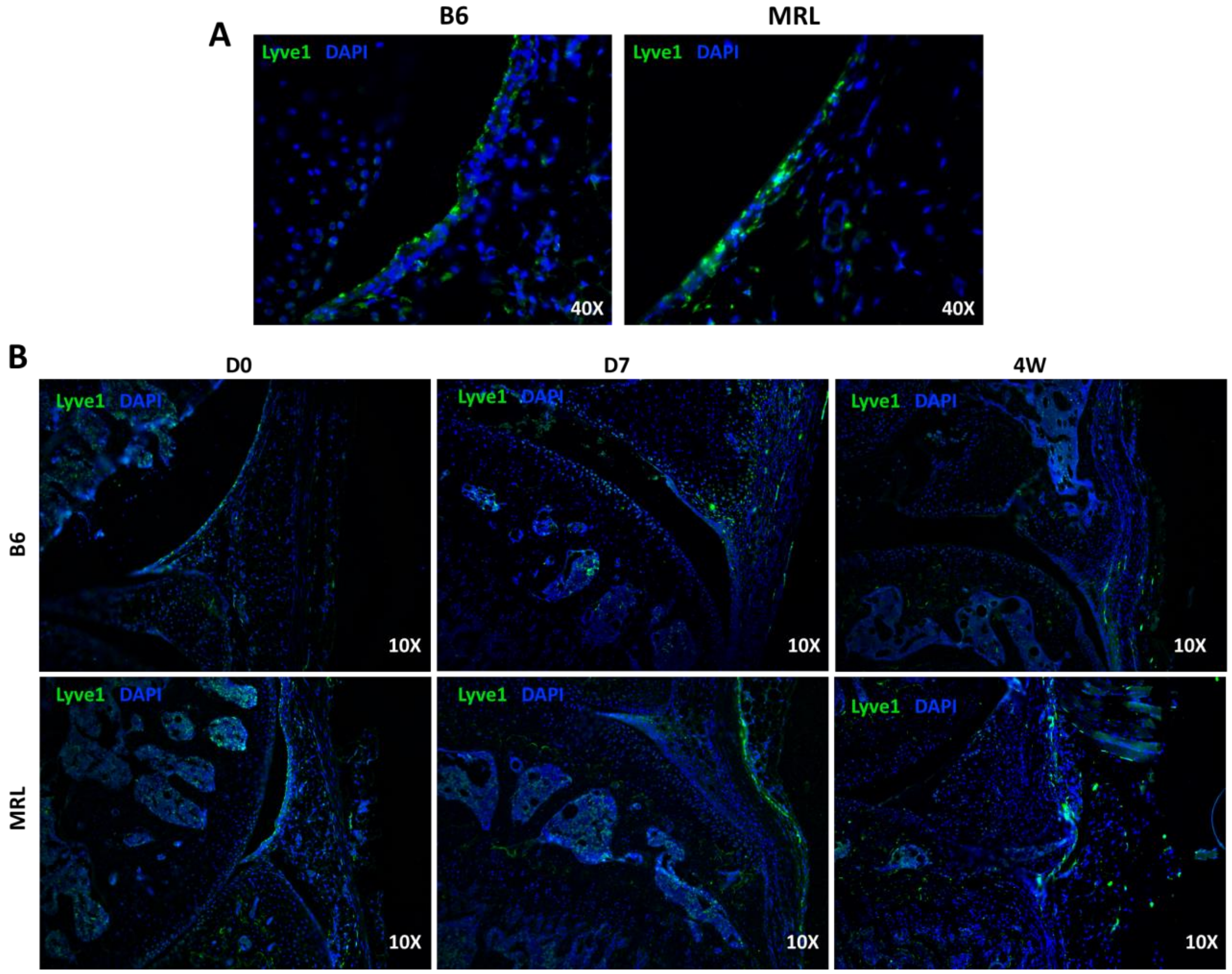

Figure S4. IHC analysis of Lyve1+ macrophages in MRL and B6 knee joints. *A) IHC showing Lyve1+ macrophages at the synovial lining, in uninjured MRL and B6 joint. B) IHC analysis of injury induced changes in Lyve1+ macrophages in MRL and B6 joints.*

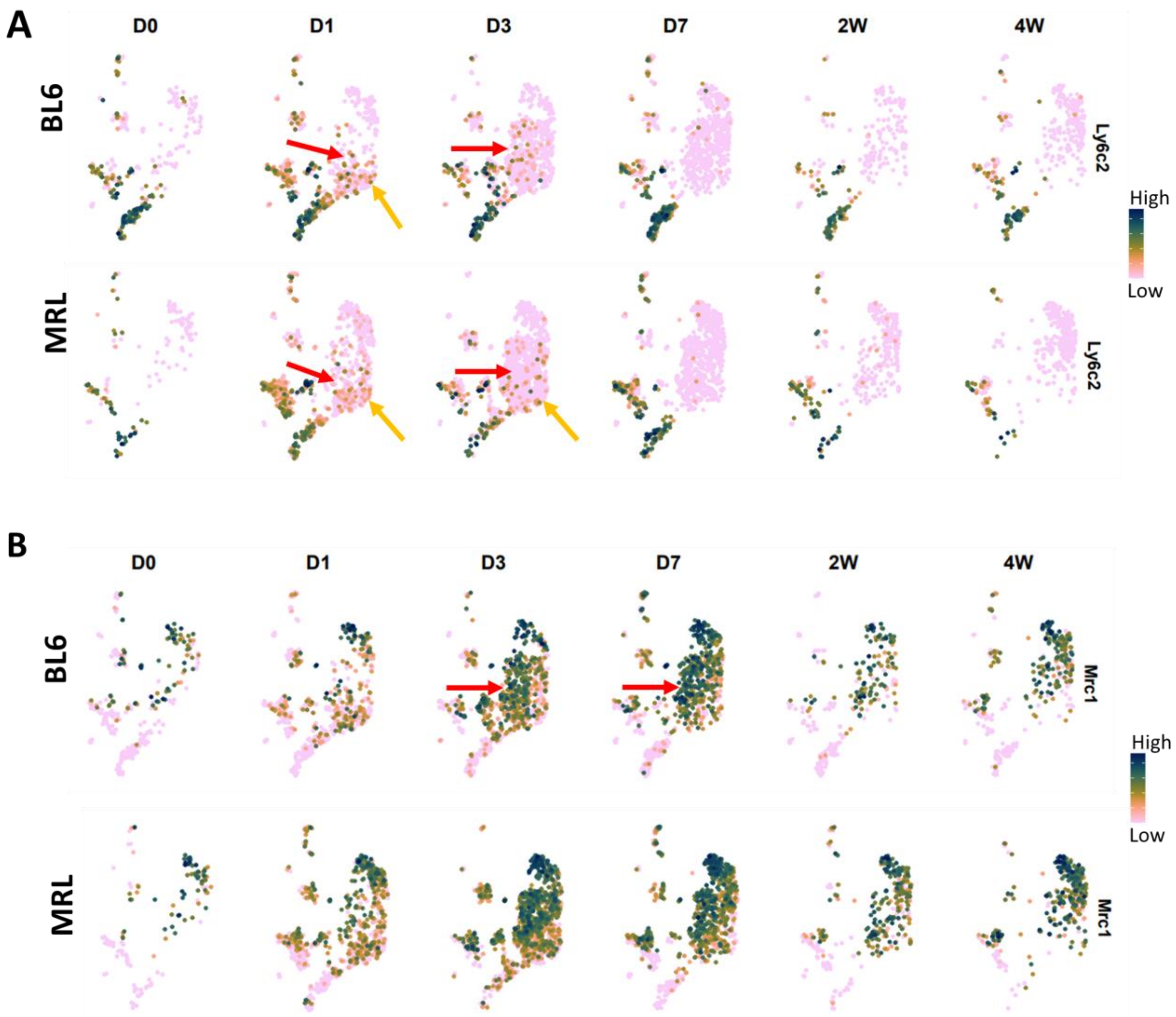

Figure S5. Ly6c2 and Mrc1 expression in macrophage clusters. *A) Ly6c2 expression was observed in Trem2⁺Fcrls⁺ macrophages (red arrow) and Spp1+Cav1+ macrophages (yellow arrow) at D1 and D3, in both MRL and B6. B) An increase in Mrc1 expression was observed D3-D7 in both strains, primarily in Trem2⁺Fcrls⁺ macrophages (red arrow).*

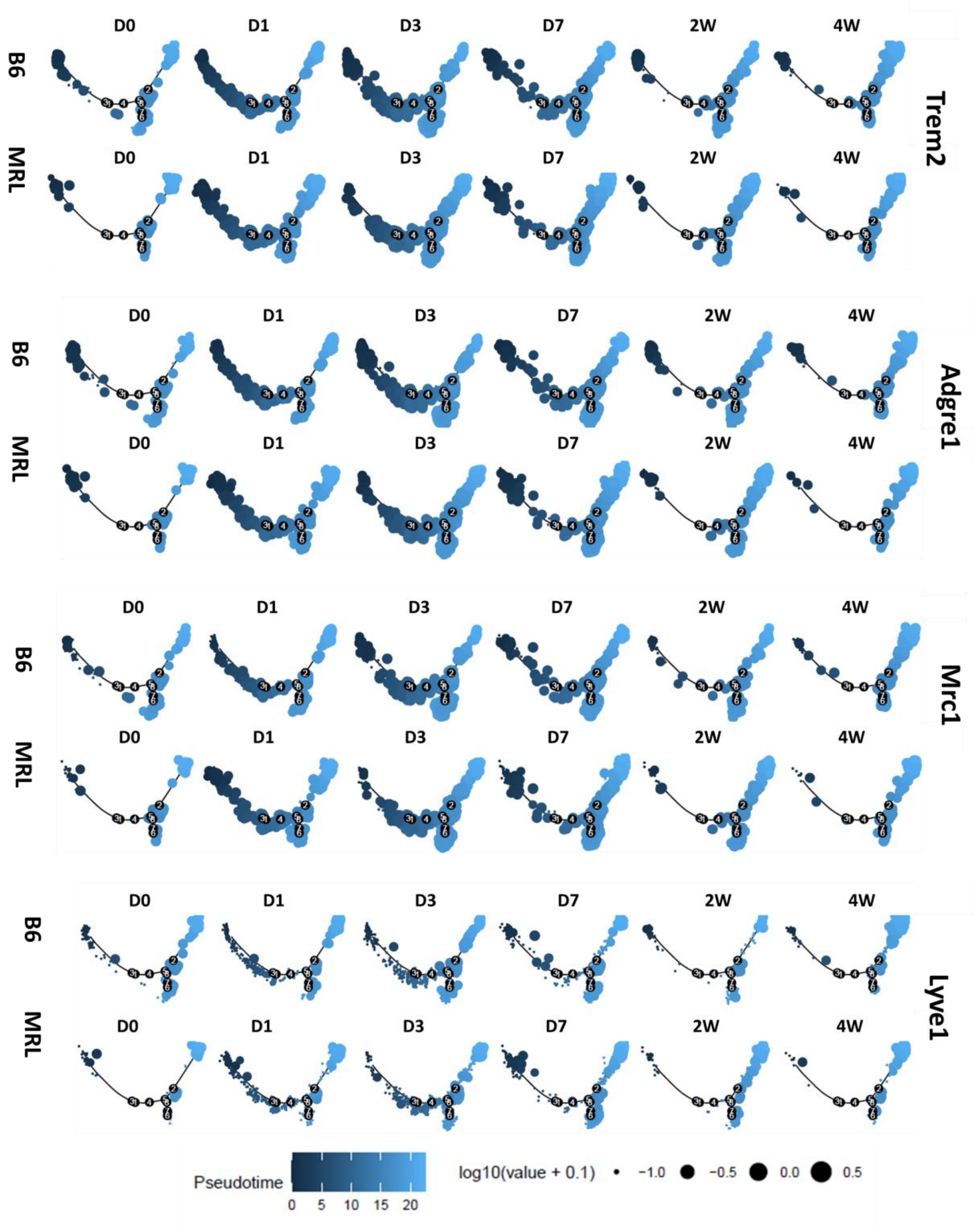

Figure S6. Gene expression on pseudo-time differentiation trajectory. *Superimposition of the expression of selected genes on the pseudo-time trajectory (colored based on pseudo-time). Circle size represents the gene expression level.*

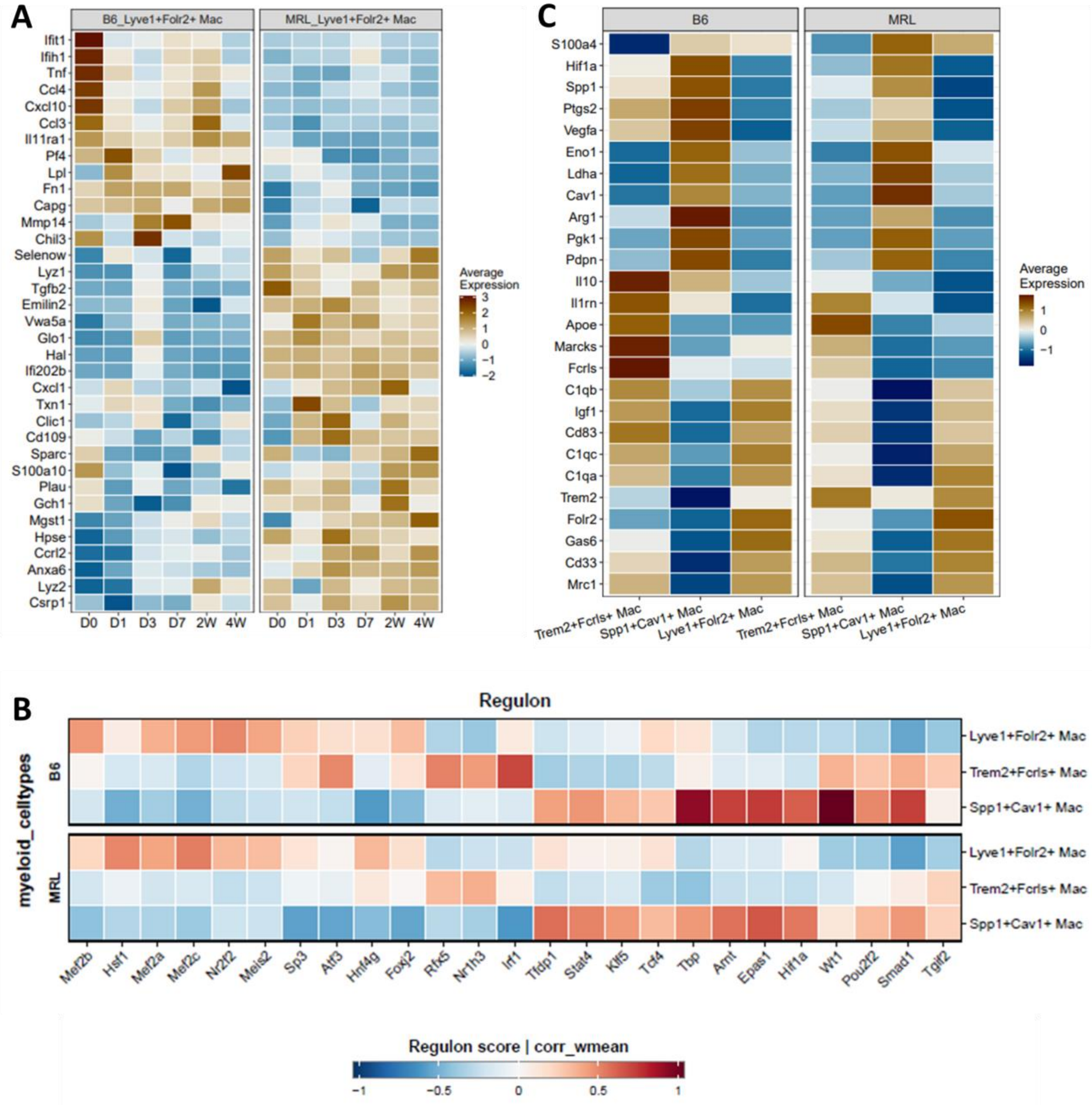

Figure S7. Differential macrophage responses observed in MRL and B6 mice after knee joint injury. *A) A subset of genes differentially expressed between MRL and B6 in Lyve1*$^{+}$*Folr2*$^{+}$ *macrophages. B) Transcription factor enrichment in various macrophage subpopulations from MRL and B6 knee joints. C) A subset of genes differentially expressed between Trem2*$^{+}$*Fcrls*$^{+}$ *and Spp1*$^{+}$*Cav1*$^{+}$ *macrophages.*
