# Supplementary material for: CD206+ Trem2+ macrophage accumulation in the murine knee joint after injury is associated with protection against post-traumatic osteoarthritis in MRL/MpJ mice
Source: PLoS One. 2025 Jan 3;20(1):e0312587. doi: 10.1371/journal.pone.0312587 (PMC11698337; doi:10.1371/journal.pone.0312587)
Supplement: Attachment — Submitted filename: McCool et al.2024_response to reviews.docx [file pone.0312587.s008.docx]

**Response to Reviewers**

**Reviewer #1:**

We kindly thank the reviewer for their suggestions and critiques and have made the following revisions and comments in response to their suggested points:

1. **Provide specific details regarding the nature of the joint injuries induced in the mice and how they correlate with human PTOA.**

We used a noninvasive tibial compression injury model that that mimics ACL rupture and subsequent PTOA development in human without surgical methodologies. This information has been added to the introduction lines 105-106. Over 15 studies have been published using this model and the originally published work as well as few other relevant studies have been cited. Our methodology section “Experimental Animals and ACL Injury Model” also briefly discusses the tibial compression injury model that was used in this study.

2. **Discuss the potential limitations or caveats of using animal models to study a human disease, such as differences in the immune system and joint physiology.**

Thank you for the suggested revision. We have added additional information on limitations of our study to the discussion lines 677-693.

3. **Include information about the synovial joints that were analyzed and their role in PTOA pathogenesis, as well as any technical details regarding the single-cell RNA sequencing methodology.**

Immune (CD45+) cells from injured and uninjured knee joints of MRL and BL6 were analyzed in this study. Using a non-invasive tibial compression injury model, we induced ACL rupture in these mice to investigate PTOA pathogenesis. Technical details of the timepoints post injury, strain, age, gender of mice is discussed in our methodology section “*Single-cell RNA sequencing and data analysis*”. In this section, we have included methods for tissue collection as well as joint dissociation for synovial cell collection. Additionally, this section includes in depth descriptions of sequencing protocols and analysis of the data files.

4. **Discuss the potential therapeutic implications of targeting the identified immune cell populations and molecular pathways in the treatment or prevention of PTOA.**

We have included additional information on potential therapeutic implications to the discussion and conclusion sections of the manuscript lines 658-662.

**Reviewer #2:**

We kindly thank the reviewer for their careful review of this manuscript. We have included revisions to the manuscript and comments to address the following points:

1. **Limited Novelty: While the study of PTOA and its molecular mechanisms is of interest, the presented findings do not provide significant new insights beyond what has already been reported in the literature.**

The enhanced healing capabilities of MRLs have been previously explored at the whole transcriptome and histopathology levels in several tissues (we have referenced Heydemann, Fitzgerald, Clark, Kwiatkowski, Rajnoch, Ward). However, no prior studies have investigated local changes to the immune response in MRL that could have contributed to the PTOA resistant phenotype, at a temporal and single-cell resolution as we have done in our study. Most current literature on MRL/MpJ used invasive surgical models such as medial meniscus displacement (Deng 2019 - PMID 31042406) and intraarticular fracture (Ward 2008 - PMID 18311808) to study PTOA progression. These invasive surgeries activate the immune system at the surgical site and may mask the true immune changes associated with PTOA progression.

In this study, we used a human-relevant, non-surgical model of ACL rupture to study PTOA progression. Using single-cell RNA sequencing technology we were able to identify injury-induced changes in various immune cell types/subtypes at an unprecedented resolution and the timepoints at which these changes occur. Our study highlighted that MRLs have increased number of a specific type of anti-inflammatory macrophages expressing *Trem2* which may have contributed to their PTOA-resistant phenotype. We have also identified several differentially expressed genes between immune cell populations within the MRL and B6 joints. The therapeutic potential of some of these genes are being investigated in ongoing studies. Additionally, we believe the single cell data generated here, while being descriptive, will greatly add to the current field of PTOA and will aid as a foundation for additional hypothesis generating work.

2. **Insufficient Contextualization: The introduction does not adequately establish the importance and relevance of PTOA in the field of medicine. It is crucial to provide a comprehensive overview of the prevalence, clinical implications, and current understanding of PTOA.**

We thank the reviewer for the suggestion. Additional information on PTOA (cause, statistics of PTOA, economic burden etc.) have been included in the introduction as follows:

*“Osteoarthritis (OA) is a painful joint disease that affects over 300 million people world-wide and is a leading cause of disability [PMID: 33671471]. Post-traumatic osteoarthritis (PTOA) is a form of osteoarthritis that develops as a result of joint injuries such as anterior cruciate ligament (ACL) rupture and meniscal tear and accounts for ~12% of all OA cases [PMID: 33671471]. This disease has a high-cost burden to healthcare systems worldwide and in the United States alone exceeds $10 billion annually [PMID:27145096]. Currently, there are no approved therapies to prevent the chronic pain and joint dysfunction associated with PTOA. Therefore, more research is needed to understand the molecular mechanisms of PTOA pathogenesis and to identify new targets for therapeutic development.”*

Additionally, we discuss the current understanding of PTOA onset and pathogenesis, as well as the current immune cell contribution to arthritis progression in other animal and injury models in the introduction.

3. **Lack of Comparative Analysis: The article does not compare the findings of the study with previous studies or existing knowledge in the field. A more thorough comparison would help to assess the significance and contribution of the current study.**

We thank the reviewer for the suggestion. Multiple studies have shown that MRL mice are resistant to PTOA. However, the cellular and molecular mechanisms contributing to this resistance phenotype is not fully understood. The goal of our study was to investigate immune-driven mechanisms of PTOA resistance in MRL by first identifying the changing population dynamics of the immune cells after injury in B6 and MRL joints. These changing populations could lead to identification of novel therapeutic targets for PTOA. Thus, we have added the following to the introduction.

*“While the ability of MRL to repair their knee joint has been histologically evaluated in several studies, primarily using invasive injury models such as medial meniscus surgery, intraarticular fracture and cartilage wounds in the trochlear groove [PMIDs: 37979958, 18311808, 18455447, 31042406], cellular and molecular interactions leading to this resistant phenotype have not yet been fully elucidated. Identifying the cellular and molecular mediators of PTOA resistance in MRL could open up new avenues for therapeutic development for PTOA. ...“*

*“To enhance our understanding of the role the immune system plays in joint repair or joint damage after injury, we employed single-cell RNA sequencing (scRNA-seq) analysis of CD45+ cells from injured and uninjured knee joints from PTOA-resistant MRL and vulnerable B6 mice by using a noninvasive tibial compression injury model that mimics ACL rupture and subsequent PTOA development in humans....”*

4. **Incomplete Methodological Description: The article lacks a detailed description of the methods used for single-cell RNA sequencing and the analysis of immune cell populations. It is important to provide sufficient information on the experimental design, data analysis pipelines, and statistical approaches employed to ensure the validity and reproducibility of the results.**

Our methodology section cites our previously published methodology for immune cell isolation from the synovial knee joint as well as describes the methodology used for single cell sequencing and analysis in detail. Please refer to methodology section “*Single-cell RNA sequencing and data analysis*”.

**5. Inadequate Discussion of Functional Implications: The article does not sufficiently discuss the functional implications of the identified differences in monocyte and macrophage subpopulations between PTOA-susceptible and PTOA-resistant mice. A more comprehensive discussion of the functional aspects would enhance the understanding of the findings.**

We have included additional information on potential functional implications of many of our findings such as increased presence of *Trem2*+ macrophages and increased expression of genes such as *Glo1* and *Vwa5a*. Although some populations such as the *Spp1*+ macrophages have been described extensively in the context of lung infections and cancer, not much is known about their role in joint or bone tissues. Further studies are required to understand their role in PTOA.

5. **Insufficient Literature Review: The article lacks a thorough review of the existing literature on PTOA, including the molecular mechanisms involved in its development or resolution. A comprehensive review would provide a broader context for the presented findings.**

Currently there is very little understanding of the molecular programs associated with PTOA onset in both a research and clinical setting. We describe the current findings of the literature associated with the immune systems response to injury, as well as resident function of these cells in a homeostatic knee joint in the introduction. Additionally, current literature discussing potential roles of different immune cells and specific genes identified in this study are included in the discussion. The primary objective this study was to understand the cellular mechanisms that contribute to the PTOA resistant phenotype in MRL through identification of local cellular changes within the joints space after trauma. Additionally, we have reviewed and cited multiple publications investigating PTOA progression in MRL as well as the healing capacity of other tissues from MRL.

6. **Lack of Clarity in Results Presentation: The presentation of results and findings in the article is unclear and lacks proper organization. The information provided should be presented in a logical and concise manner to facilitate understanding.**

We apologize for the lack of clarity. We have modified the introduction, discussion and conclusions significantly to facilitate the understanding of the relevance of our findings. We understand that single-cell data from longitudinal experiments such as ours can be overwhelming but, we believe the data generated here would add great value to the community and could be hypothesis generating for others working in the field. The results section provides a top-down view of the cell types present, starting with a broad characterization of the immune cell populations as a whole. Within the Results section, Sub-section 1 discusses the baseline populations present prior to injury and after injury. We then moved forward to discuss the largest population shifts seen in the data (neutrophils and mono-macs). Each of the following sub-sections is organized to further discuss specific subpopulations of immune cells with unique transcriptomic profiles in response to injury.

7. **Incomplete Discussion of Limitations: The article does not adequately address the limitations of the study, such as sample size, potential biases, or the generalizability of the findings. It is important to acknowledge and discuss these limitations to provide a balanced interpretation of the results.**

Thank you for the suggestion. We have added study limitations to the discussion section.
