# Supplementary material for: CD206+ Trem2+ macrophage accumulation in the murine knee joint after injury is associated with protection against post-traumatic osteoarthritis in MRL/MpJ mice
Source: PLoS One. 2025 Jan 3;20(1):e0312587. doi: 10.1371/journal.pone.0312587 (PMC11698337; doi:10.1371/journal.pone.0312587)
Supplement: Attachment — Submitted filename: Response to Reviews.docx [file pone.0312587.s009.docx]

Reviewer #3: This study mainly involved animal model construction and single cell sequencing analysis of synovial joint tissue. We suggest that more in vivo histological tests and potential mechanisms be explored.

We kindly thank the reviewer for their suggestion. Although this study mainly describes findings based on single cell sequencing analysis, we have included several flowcytometry and histology data verifying our findings (Figure 2 B-D, Figure 3 A-B and Figure 7). Indeed, additional mechanistic experiments are worthy of doing, however we believe the single cell data generated here would add great value to the community and could be hypothesis generating for others working in the field. We appreciate any feedback that can improve this work and make it useful to the community, but it is impossible for new mouse experiments to be initiated at this point due to lack of funding.

Reviewer #4: Here, the authors have revised their manuscript focused on the differences between B6 mice (which are highly susceptible to PTOA) and MRL superhealer mice (which show protection), with respect to synovial immune cell populations. Overall I think this is a valuable contribution to the field given that our understanding of why some injured people go on to develop PTOA (often rapidly) and why some don't, is very limited. Studying natural differences in regenerative capacity within mice strains is a good approach for tackling this conundrum.

I believe that the authors have sufficiently addressed the comments from the first round of review, and improved the manuscript. As such, I recommend it for acceptance. Here I am providing some additional comments for the authors to take into consideration.

1) A major hurdle to studying intra-articular/synovial cells, especially immune cells, is harvesting truly representative "joint resident" cells and avoiding contamination from other sources - the most notorious being the bone marrow, given how dense with immune cells it is. Here the authors have painstakingly tried to overcome and address this hurdle, which I applaud them for. Having bona fide intra-articular cells in a single cell dataset is critical for drawing conclusions about the nuanced populations that reside in or infiltrate the joint space in homeostasis and after injury.

We kindly thank the reviewer for their positive feedback.

2) Specifically, relating to potential BM neutrophil contamination, line 382 states "to determine if neutrophils in our digests were bone marrow-derived..". In the future, it could be worth considering staining BM/circulating neutrophils prior in an inducible fashion (whether with a creERT2 or fluor-labeled in vivo delivery prior to harvest, to truly distinguish BM/circulating neutrophils from bona fide intra-articular cells. This is at the forefront of the field and would be a worthwhile pursuit, as it has not been done before (to my knowledge).

We kindly thank the reviewer for their suggestion. In future studies we will try to better label neutrophils to truly distinguish BM/circulating neutrophils from bona fide intra-articular cells, this is a current limitation and we have stated this in the discussion now.

‘Furthermore, we currently can’t distinguish between BM/circulating neutrophils from neutrophils that have infiltrated the joint tissue, but future lineage tracing experiments will aid in this endeavor.’

3) Minor suggestion for future studies - we and others have found that dramatically slowing the rate of perfusion helps to prevent bursting of delicate narrow capillary beds in peripheral tissues, like synovium. Here the authors perfused at 10mL/min - I would suggest slowing this to 200mL/h.

We kindly thank the reviewer for their suggestion. In future studies, we will try slowing down the rate of perfusion.

4) In lines 438-452, there may be some confusion about cluster numbers that you are referring to. Please double check this to ensure it's correct throughout this section.

We kindly thank the reviewer for pointing out this inconsistency. We have adjusted the cluster number for Spp1+Cav1+ subpopulation (changed cluster 2 to cluster 3) in this section of the manuscript. We have also reviewed the remainder of the text and ensured there are no more misnumbered clusters.
